# How Amino Acids and Peptides Shaped the RNA World

**DOI:** 10.3390/life5010230

**Published:** 2015-01-19

**Authors:** Peter T.S. van der Gulik, Dave Speijer

**Affiliations:** 1 Centrum Wiskunde & Informatica, Science Park 123, 1098 SJ Amsterdam, The Netherlands; E-Mail: Peter.van.der.Gulik@cwi.nl; 2 Department of Medical Biochemistry, Academic Medical Center, University of Amsterdam, Meibergdreef 15, 1105 AZ Amsterdam, The Netherlands

**Keywords:** coevolutionary theory, RNA world, prebiotic chemistry, evolution

## Abstract

The “RNA world” hypothesis is seen as one of the main contenders for a viable theory on the origin of life. Relatively small RNAs have catalytic power, RNA is everywhere in present-day life, the ribosome is seen as a ribozyme, and rRNA and tRNA are crucial for modern protein synthesis. However, this view is incomplete at best. The modern protein-RNA ribosome most probably is not a distorted form of a “pure RNA ribosome” evolution started out with. Though the oldest center of the ribosome seems “RNA only”, we cannot conclude from this that it ever functioned in an environment without amino acids and/or peptides. Very small RNAs (versatile and stable due to basepairing) and amino acids, as well as dipeptides, coevolved. Remember, it is the amino group of aminoacylated tRNA that attacks peptidyl-tRNA, destroying the bond between peptide and tRNA. This activity of the amino acid part of aminoacyl-tRNA illustrates the centrality of amino acids in life. With the rise of the “RNA world” view of early life, the pendulum seems to have swung too much towards the ribozymatic part of early biochemistry. The necessary presence and activity of amino acids and peptides is in need of highlighting. In this article, we try to bring the role of the peptide component of early life back into focus. We argue that an RNA world completely independent of amino acids never existed.

## 1. Introduction

The idea of an independent RNA world without oligopeptides or amino acids stabilizing structures and helping in catalysis does not seem a viable concept. On the other hand, the idea of catalytic protein existing without RNA storing the polypeptide sequences, which have catalytic activity, and organizing the production of these sequences, also does not seem a viable concept. Here we argue for a “coevolutionary” theory in which amino acids and (very small) peptides as well as small RNAs existed together and where their separate abilities not only reinforced each other’s survival, but allowed life to more quickly climb the ladder of complexity. Essential for our approach is the following: Starting with small molecules (easily) derived from prebiotic chemistry, we will try to reconstruct a possible history in which every stage of increased complexity arises from the previous more simple stage because specific nucleotide/amino acid (RNA/peptide) interactions allowed it do so. These interactions occurring during pre-biological evolution would have been selected for molecular functionality, as exemplified by “*co-catalysis*” and protection from breakdown. We are well aware that some parts of the reconstruction are more speculative than others: When trying to piece together a possible scenario for the origin of life this cannot be otherwise. Before introducing such a coevolutionary theory, however, we have to clarify the history of the term and what *we* mean by it.

## 2. The Original Coevolution Theory of Genetic Code Origin

This original theory used the term “coevolution” somewhat differently from how we will define it here. Its history derives from the fact that after the original synthesis of Alanine, Glycine, and Aspartic acid by Miller in 1953 [1], the optimism regarding this kind of prebiotic synthesis was so great that all 20 canonical amino acids were expected to appear in such experiments. To quote: “one can say that 13 of the 20 amino acids in proteins can be formed ... Cysteine was found in the photolysis of CH_4_, NH_3_, H_2_O and H_2_S [2]. The pyrolysis of hydrocarbons ... leads to phenylalanine, tyrosine, and tryptophan. This leaves ... lysine, arginine, and histidine. ... There is no fundamental reason the basic amino acids cannot be synthesized, and this problem may be solved before long.” [3]. The genetic code would then have evolved in an environment containing all of them. As a first aside, we have to mention that Miller presumed the existence of a reducing environment, while at present most geochemists think a neutral or even slightly oxidizing atmosphere more likely. However, even under these conditions several amino acids (even quite complex ones) can still be formed, see, e.g., comments in [4]. Opposing this idea of rapid evolution of a full amino acid complement, coevolution theory argued instead for a coevolution of the growing number of amino acids in the code repertoire, and the growing powers of biosynthesis making new ones (e.g., Tyrosine or Asparagine). In effect, code and metabolism would be evolving together [5,6]. More code allows more (sophisticated) metabolism, and an extended metabolism in turn extends the code (“code” here meaning “number and kinds of amino acids specified by codons”). Originally, the most important aspect of Wong’s coevolution theory was that the larger amino acids of the canonical set of twenty did not appear in prebiotic synthesis experiments, and thus were surmised to have come about as the result of biological activity. Nowadays the aspect of biosynthesis on the tRNA [7,8,9] is seen as the much more salient aspect of the theory. This illustrates that the idea of a growing coding repertoire interacting with a simultaneously growing repertoire of biosynthetic products seems completely accepted. Here we will extend and build on this aspect of the theory, trying to reconstruct details of the molecular interactions involved.

## 3. What Do We Mean by “Coevolutionary” Theory Here?

According to Szostak, short peptides with several acidic residues could have been very important in protecting short RNA molecules against degradation by Mg^2+^ ions [10]: See below. According to Noller, interaction with RNA molecules, and stabilizing certain RNA conformations, could have been the first function of short coded peptides [11,12]. In present-day biochemistry, Arginine and Lysine take care of most of the interactions with RNA. However, we should not forget that Glycine, which is arguably an “older” amino acid than Arginine and Lysine, also has the capacity to interact with RNA by way of its nitrogen atom, which ends up in the peptide-bond group (please note here that the genetic code structure possibly started out with the following four-column structure: Hydrophobic, small, cation-binding, anion-binding) [13]. In [13], the functional relation between Arginine and Glycine was not recognized (yet); it is pointed out in [14], however. Functions as envisioned by Noller and by Szostak, performed by short peptides formed by non-coded synthesis (mainly consisting of Glycine, Alanine, and Aspartic acid, all found in the very first Miller synthesis) could have been crucial for the earliest steps of the RNA world. The GlyGly dipeptide, possibly formed on montmorillonite [15], the mineral enhancing condensation reactions under Salt-Induced Peptide Formation (SIPF) [16] wetting-drying cycle conditions, could itself have been crucial for the production of short, Aspartic acid-containing, Mg^2+^-binding, RNA-protecting peptides. Peptides can form ([17] and references therein) when a complex is formed in which Cu is bound to one amino acid via coordination with its N and O sites, to another amino acid by its carboxylic group, while also interacting with a Cl^−^ ion, derived from NaCl, with Na^+^ (when occurring with an underpopulated water shell) acting as a dehydration agent. Under such circumstances (in which evaporation of water is crucial) the presence of glycine or GlyGly has been shown to speed up dimer formation and oligomerization of other amino acids. One could say that the GlyGly dipeptide is responsible for further peptide bond formation under SIPF conditions [4,18].

Of course, such an interaction is a far cry from *coded* synthesis of this dipeptide, but the possibility that coded synthesis originated as homopolymerization of Glycine (or even just dimerization of Glycine), taking into account the catalytic effects of GlyGly [18] (see below), should be taken seriously. We will try to show that all, or most, steps along the road to protein did have selective advantage. It is clear that the system did not arise full blown with long heteropolymers of amino acids already aiding at the start of the evolutionary process towards making them.

In a much later stage, peptides were made from Glycine, Alanine, Aspartic acid, and Valine. At least, this is a viewpoint held by some leading scientists working in the field [13,19,20]. The cellular factory making such peptides was the early ribosome. We now know that when the rRNAs are fragmented, the ribosome still forms [21,22,23,24]: The connection of all the parts in the complete large rRNA seems mostly a matter of co-regulation of their synthesis, not essential for function. Many small rRNAs can still make a functioning ribosome. However, the presence of rProteins *is* essential for ribosome formation [25,26]. Possibly, a ribosome in the early stages of evolution consisted of many rRNAs stabilized by a few small peptides containing glycine, alanine, aspartic acid and/or valine, essential for the structure of the nucleoprotein particle. The ribozyme was the superior catalyst then (but GlyGly and/or other peptides containing glycine, alanine, aspartic acid and/or valine could already have been crucial for function at this stage).

Thus, what we mean with “coevolutionary” is that evolutionary lengthening of peptides and nucleic acids happened simultaneously. Such “mutual assistance” is also already mentioned in an article by Carter and Kraut [27], however they model with a beta sheet; a *two-stranded* peptide, which of course already exemplifies a rather “late” development in early evolution: Where could such a complicated molecule have come from? Here, we try to explain the emergence of both longer RNAs and peptides by going back much further. We will try to show that amino acids and peptides were essential for the existence of RNA; and that the ribosome could have begun as a nucleopeptide particle, with small RNA molecules being coordinated and protected by amino acids and small peptides. The big challenge for investigators of the early RNA world is to unravel the steps between the first spontaneous GlyGly formation and the stage in which a primordial ribosome is producing relatively small peptides containing glycine, alanine, aspartic acid and valine. Once this last stage is reached, further evolution towards last universal common ancestor (LUCA) can be envisaged more easily. We will discuss some remarkable issues concerning this early evolution of the RNA world next.

## 4. The Ubiquitous Presence of “Prebiotic” Amino Acids

Amino acids are all around. They are produced in outer space and rain upon earth with meteorites [28]. They are also produced by the combination of lightning and volcanism [29], mostly ending up in the sea. Possibly, they are also produced by deep-sea smokers arriving in the oceans along that route [30]. Last but not least, they can be formed on pre-biotic earth under reducing [31], as well non-reducing conditions [17]. Summarizing, before the advent of organisms using amino acids, they would abundantly accumulate in the earth system.

## 5. The Presence of Peptides

Abundant presence of amino acids on earth is one thing, peptide formation quite another. In this context, especially the work of Rode and colleagues seems to be enlightening. Some dipeptide formation can occur by comet impact [32]. The catalytic action of glycine and GlyGly, formed according to the mechanism described above, allowing more efficient formation of dipeptides from mixed glycine, aspartic acid, valine, and at later stages other amino acids in SIPF reactions, could result in important positive feedbacks [18]. Interestingly, SIPF reactions, in which depleted hydration shells around sodium ions allow the dehydrating condensations of peptide formation to occur without biochemistry [16], are further improved by montmorillonite clay [15]. Also stabilizing effects of surfaces of clay minerals, like hectorite and montmorillonite, were shown to be important in protecting larger compounds against hydrolysis and other forms of decomposition. To conclude: In an environment full of amino acids in atmosphere and ocean, specifically where sea and land meet, such circumstances could occur (as exemplified by rhythmic patterns of ebb/flood, day/night, sun/rain, and geyser activity) that favor the stable formation of a variety of dipeptides, tripeptides, and even longer oligopeptides, especially if the surface layer of sediment is montmorillonite.

## 6. The Protection of RNA

Above we mentioned the possible role of (aspartate-containing) peptides in protecting RNA from breakdown catalyzed by magnesium ions [10]. This observation again points to a coastal environment with stretches of montmorillonite clay as one of the possible places for stages in the birth of “life”. Montmorillonite not only could have boosted the Aspartic acid content of such peptides (the clay binding the acid groups of the amino acids) but it most likely improved RNA oligomerization as well. Even if the primary production site of such highly vulnerable RNA oligonucleotides was somewhere else, e.g., a prebiotic intermountain dry valley, receiving basic run-off materials from olivines and borates minerals [33], the montmorillonite/sea-water coastal environment (sea-water because of the necessity of the deprived water shell Na^+^ ions essential for the SIPF reaction) could have functioned as a conservation pocket for RNA molecules washed into montmorillonite-bottomed pools. We touch upon a well-known general problem here: The fact that specific parts of scenarios invoked to explain the origin of life can only function in radically different milieus, with completely different chemical parameters. However, prebiotic earth probably was very rich in quite different microenvironments with many points of contact. Such frontiers must have been essential during the development of life. For a more general discussion of these aspects, see [34].

## 7. Closing the Loop

We now come at a crucial and, we have to admit, somewhat theoretical juncture: GlyGly production from glycine should have been a pretty simple function for RNA to come up with, if it improved survival of the RNA, and thus would have been selected for. To put it succinctly: RNA produces GlyGly; GlyGly produces Aspartic acid-containing oligopeptides; Aspartic acid-containing oligopeptides protect RNA. In this way, a positive feedback loop creating RNA could have arisen. Thus, coevolution is illustrated by the presumption that RNAs could not persist without peptide protection, that very short (very early) peptides were made more abundant by RNA producing them, and that they co-evolve forming longer RNAs and peptides. This would constitute an RNA/peptide world of ribozymes and short oligopeptides. These oligopeptides had RNA protection functions (DADVDGD being the obvious ancestor sequence of the universal RNA polymerase active site sequence NADFDGD; see [35] for the evolution of this specific stretch of amino acids, central in all cellular life), and possibly carbon storage functions (e.g., AAAGAAA would be an appealing primordial storage compound) as well as catalytic functions: e.g., GlyGly [18], and ValAsp [36]. Though the full power of polypeptides (depending on large structures with alpha helices and beta sheets) can only be developed in a more complex RNA world with larger genetic storage capability, it is not that difficult to see it developing from such a starting point. A few important complicating aspects have to be mentioned, as we have only described *selection* functioning at the level of *individual* molecules at this stage. Presumably “collective evolution” [37] could also start functioning at the level of *communities* of molecules. A possible example of influence “in trans”: We start out with the GlyGly producing RNA benefiting from the protection by Asp-containing oligopeptides that are able to sequester Mg^2+^ ions. These Asp-containing oligopeptides are in turn produced by GlyGly. How other RNAs and (di)peptides would influence further evolution we can only speculate about. A possible scenario will have slowly developing communities of ribozymes, amino acids and small peptides in which more complex mutual dependencies could evolve. Thus we would get much more variation in molecular communities which would enhance the speed of evolution.

## 8. Yarus’s Miniscule Ribozyme

The smallest ribozyme known so far is only five nucleotides long [38,39]. Three of these nucleotides represent the Watson-Crick binding pair complements of the 4-nucleotide substrate. Two of its nucleotides together with one nucleotide of the substrate form a catalytic site, binding activated amino acid, as beautifully illustrated in Yarus’s paper (Figure 4 in [40], p. 2904). In view of our coevolutionary argument, it is highly relevant that the reaction catalyzed by this smallest ribozyme active site known, is aminoacylation. These tiny ribozymes could thus have been making GlyGly, in turn making, e.g., AspGlyAsp, in the process stabilizing themselves. However, they did not recognize amino acid side chains as such: Instead they recognized phosphate-and-amino-acid-backbone parts of *activated* amino acids. One could describe this as catalyzed non-coded amino acid dimerization. GlyGly and other dipeptides are produced. Viewed like this, the work of Yarus and colleagues represents a landmark discovery: Describing the *first* ribozyme synthetase. We have to especially highlight another aspect as well: One of the substrates of the tiny ribozyme is an *activated* amino acid. Could amino acids already have been directly activated by ATP at this stage? This would imply that ATP has been the energy currency of life from a very early stage onwards, and that all the research done during the past decades on alternative activation mechanisms is interesting from a chemical point of view, but possibly irrelevant from the viewpoint of the history of life on earth. Of course adenine, which can be formed by polymerization of cyanide [41], is not too difficult to envision as a prebiotic product. As far as ribose is concerned, Benner and co-workers have reported that borate can save it from “becoming asphalt” [33,42]. On top of this, Di Mauro and co-workers reported that phosphate-containing minerals have the power to produce nucleotides [43]. The combination of these studies hint at a solution for one of the great problems in origin of life studies: The source of prebiotically produced ATP, if any.

Having loaded pentanucleotides available, however, is only a first step in getting non-coded ribozymatic dipeptide synthesis going. Here it is important to be aware of the active participation of amino acids in protein synthesis. It is *the amino group* of the loaded amino acid, which is of course involved in the actual reaction. The peptidyl transferase centre (PTC) of the ribosome is orienting this amino group such that it can efficiently attack the peptide-tRNA connection of the peptidyl-tRNA, during protein synthesis. In principle one would expect that montmorillonite also has the potential to align two loaded RNAs in the correct orientation such that peptide bond formation could occur. The conformational change of the tRNA upon release of the amino acid could be crucial in making the process directional. Such a scenario would couple the *dynamic* nature of the tRNA (already pointed out by Woese in 1973 [44]), with the necessity of *one-way directionality* in translation [45]. Possibly a conformational change allows the empty tRNA to leave, while also creating a conformational alteration in the peptidyl-tRNA opening up space to accommodate an incoming loaded tRNA on the montmorillonite surface. Seen in this way, tRNAs are more central to the process than mRNAs and rRNAs.

## 9. Shimizu’s Highly Remarkable Experiments: From Non-Coded to Coded Synthesis?

Twenty years ago, Shimizu reported that an RNA stem-loop (the “C4N structure”), with two dangling ends, a 5' GCC terminus and a 3' UCCA terminus, was loaded specifically with Glycine, in the presence of ValAsp and activated amino acids [36]. Furthermore, a 3' ACCA terminus could be loaded specifically with Alanine if the 5' end was AGC or Valine if the 5' end was GAC. No such relations were reported by Shimizu for middle-U anticodons and Aspartic acid. However, the recognition of Glycine, Alanine, and Valine by very simple small RNA motifs (representing their respective anticodons) is already quite staggering, and might reflect the emergence of coding. By acquiring a GCC anticodon at the 5' end, and a discriminator U followed by a CCA-terminus at the 3' end, many different short RNA stem-loops could evolve the possibility to be *specifically* loaded by Glycine, at least in environments containing ValAsp and activated Glycine. ValAsp could thus be seen as the appearance of the first “protein” aminoacyl tRNA synthetase (aaRS) with the first anticodon of the genetic code (GCC). The “synthetase” is of course still non-specific: The “tRNA” itself is performing the specific selection of amino acid. If one of the short RNA stem-loops fitted into the combined montmorillonite phosphotransferase system, the amount of Glycine entering oligomerization could be boosted. This would further increase concentrations of, e.g., GlyGly and AspGlyAsp.

It is strange to notice that follow-up experiments (even at the level of reproducing the results obtained, let alone extending them) seem not to have been performed, as far as we know. This becomes even more puzzling when we consider the momentous step the Shimuzu experiments seem to illuminate: The possible origin of coded synthesis.

## 10. Di Giulio’s Insight: tRNA Was a Dimer

In a string of publications, Di Giulio defended his remarkable suggestion that tRNAs originated from the duplication of a “hairpin gene” [46,47,48,49]. This scenario would help explain how the observed present-day connection between the codon/anticodon triplet code and the “second genetic code”, partially encoded by the so-called discriminator nucleotide [50,51,52,53,54], relating specific tRNA determinants to specific aminoacylation by the different aaRSs could have evolved from relatively small peptides (see below and next paragraph). Apart from this basic observation, Di Giulio also claims a non-monophyletic origin of current tRNAs as illustrated by the assembly of two minigenes codifying for different RNA hairpin structures in *Nanoarchaeum equitans*, which he thinks reflects the primordial configuration [55]. He also interprets the existence of permutated tRNA genes in *Cyanidioschyzon merolae*, as ancestral [56]. However, both seem to be examples of derived, acquired characteristics [57].

**Figure 1:**
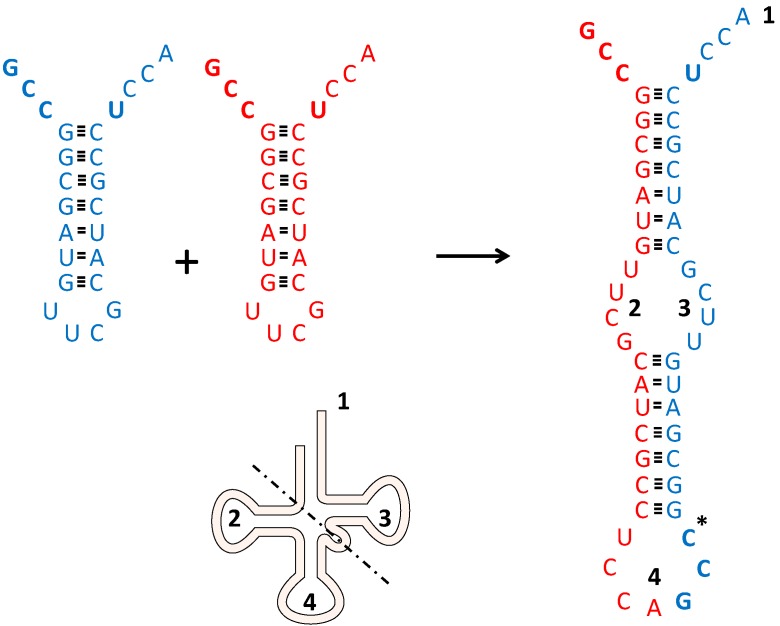
How to get a tRNA precursor. Two (identical) hairpin-loop structures (red and blue) can form one extended RNA molecule upon unfolding. Regions in this precursor are compared to a schematized version of a present day tRNA. 1: CCA acceptor stem; 2: D-arm; 3: T-arm; 4: Anticodon containing loop. The dotted line indicates the schematized separation between acceptor-TψC stem-loop and anticodon-D stem-Biloop [52]. The anticodon and the discriminator nucleotide U are shown in bold face. Hydrogen bonds are shown as stripes. * In the anticodon containing loop further insertions and deletions are needed. Here two identical molecules (accepting glycine) are depicted, however the extended molecule can also be formed by slightly different non-identical molecules. For further details see text.

We could thus envisage two relatively small hairpin sequences basepair, forming a primitive tRNA precursor. If we begin with two “Shimizu” RNA stem loop molecules (C4N structures), each with a 5' anticodon-stem loop stem-discriminator nucleotide-CCA 3' structure (see Figure 1) and allow them to basepair after “opening up” their original stem loops, eventual ligation on one side of the molecule (where the differently colored sequences meet in Figure 1) would lead to a new RNA species. This molecule has both an anticodon in the middle and a discriminator nucleotide-CCA at its 3' end, and would start resembling a modern tRNA. Three major aspects should be stressed here.

(1) Much less extensive RNA molecules are necessary than was thought previously to produce rather large RNA structures. Base pairing could lead to something resembling a primitive tRNA starting out with relatively small RNA molecules. The same general point (several small RNA molecules making up a larger RNA structure) of course holds true for rRNA. That this arrangement can work is nicely illustrated by the mitochondrial ribosomal RNA, which is now encoded in scrambled gene pieces in *Chlamydomonas reinhardtii* mitochondrial DNA [21]. This could be seen as an evolutionary regression to a situation resembling the original state: A possibility precisely because this is how the system originated (compare the related phenomenon of the reduced tRNA set functioning in mitochondria).
(2) The scenario described could explain the perplexing fact that originally the anticodon should be close to the CCA acceptor stem to allow acylation with the correct amino acid while it is now only found at the other side of the tRNA: In the beginning *both* ends had an anticodon! The anticodon close to the acceptor side chooses Glycine, Alanine, or Valine. The anticodon in the middle becomes available for mRNA.
(3) This scenario should be distinguished from another popular model regarding tRNA evolution: The one in which “tRNA” started out as a small CCA acceptor only, which would be aminoacylated according to the second genetic code, with the current genetic code appearing at a later stage upon extension of the acceptor minihelix with an anticodon stem-loop (see e.g., [53]). It should be stressed that the two domains (acceptor-TψC stem-loop and anticodon-D stem-Biloop, which is taken as a later addition) do *not* coincide with the two original regions of the “Di Giulio dimer”, as can be clearly seen in Figure 1. In our model the acceptor stem is not “older” than the anticodon loop and the link between the genetic code and the second genetic code “read” by the aminoacylating domain of the synthetases is not tagged on afterwards, but present because they are sterically together from the beginning. This explains the specificity of the different aaRSs even though they must have started out too small to bridge the distance to the anticodon loop.

## 11. Towards a More Unambiguous “Code”

Up till now we have envisaged coordination occurring on montmorillonite, with a hypothetical community of four different tRNAs, several mRNAs and some very short peptides made up of Glycine, Alanine, Aspartic acid and/or Valine collaborating to achieve a highly primitive and presumably very inefficient, but coded (!) protein synthesis. GlyGly is most likely produced rather unambiguously. ValAsp, however, is produced in a less straightforward way, because the tRNA with a middle-U anticodon is loaded with different kinds of amino acid depending on their relative amino acid concentrations. Two ways are available to achieve a higher abundance of Aspartic acid with the middle-U loading: (1) enhancing metabolism transforming Glycine, Alanine and Valine into Aspartic acid (most likely via pyruvate and oxaloacetate from a very early stage); (2) producing the first different aaRSs (variations of the peptides present in our scenario). Such “variations of Glycine, Alanine, Valine and/or Aspartic acid peptide synthetases” would first catalyze (almost) unselective tRNA loading, but then selective middle-U tRNA loading with acidic amino acids would have to develop. In this respect it is telling that Rodin and Ohno suggested that aaRSs originated as couples of core synthetase proteins, coded sense and anti-sense on the same nucleic acid double strand [58]. Ribas de Pouplana and Schimmel [59] showed that Class I and Class II aaRSs originally formed a complex around one tRNA, the synthetases fitting exactly around a single tRNA molecule. Shaul *et al.* [60] showed how such synthetase proteins could evolve step-by-step towards more and more unambiguous recognition of amino acids. The first two couples of synthetases could respectively distinguish aliphatic (Valine, Isoleucine, Leucine) from small (Serine, Proline, Threonine) amino acids and the acidic amino acid Glutamic acid from its counterpart Aspartic acid. Glycine and Alanine would in these early times most likely still be aminoacylated to their tRNAs by a Shimizu-like mechanism. Li *et al.* showed that 120–130 residue fragments of aaRSs of both Class I and Class II (which remain when all later additions are removed from the “urzyme”) already catalyse tRNA acylation at a high rate (both urzymes function at ~60% of present-day efficiencies) [61]. Thus such simple ancestral aaRS urzymes might have pre-dated modern ones, and already had large catalytic repertoires with sense/antisense coding. They exemplify intermediate steps in protein-tRNA co-evolution. Before describing the possible development of an extended amino acid repertoire to make up more advanced protein however, we should now first turn to the introduction of rRNA and the primordial ribosome. It will quickly become clear that extending small peptide synthetases and originating catalysts to speed up metabolism is out of the question without a primitive ribosome. The stabilizing, protective aspect of Aspartic acid containing peptides, which was extensively discussed above, would have reached its limit without further developments.

## 12. Reconstructing the Primordial Ribosome, Tunnel and All

Fox *et al.* suggest [62] that without the evolution of an internal cavity of a protoribosome, longer oligopeptides will not be produced. Here we need the pre-rRNA in the form of a ribosomal tunnel (a precursor of the current exit tunnel) to enter our set of early life mechanisms. As such, the presence of peptides in the tunnel would increase both lifetime and length of oligopeptides in such a protoribosome. However, another role of the pre-rRNA possibly was speeding up regulated, more specific, termination. Until now, termination has probably been dependent on (mostly random?) water molecules, engaging in spontaneous hydrolysis. This is quite likely: The existence of hydrolysis is the reason that we do not expect long RNAs to exist at all under prebiotic conditions. But the random process could have been relatively slow and unregulated. Having “pre-rRNA” guiding the water molecule to cut the peptide from the tRNA, e.g., whenever it takes too long before a new loaded tRNA occupies the primordial A-site, is both speeding up processes and adding specificity. Life is gathering momentum in this scenario. There are a few snags, however.

First of all, we seem to need quite large rRNA-like molecules to build such a cavity, while peptide catalysts still seem to be limited to relatively small oligopeptides. Secondly (see below), when looking at reconstructions of the ancient PTC, we seem to find absolutely no traces of the involvement of small peptides or amino acids here [63,64,65]. So did the RNA world develop independently of amino acids and small peptides after all, at least in as far as the ribosome is concerned? When discussing the evolution of the ribosome, especially the work of Williams and colleagues has to be considered [63,64,65]. In what they aptly call “molecular paleontology”, they show the ribosome to be made up of layers of increasing complexity and are able to reconstruct the most ancient part of the large subunit. The oldest parts of the ribosome seem to be the PTC and exit tunnel. Later on coding (associated with the cooption of an independently evolved small subunit RNA of unknown function or a small rRNA evolving “*de novo*”) and translocation capacities seem to have been progressively built up, while also extending the exit tunnel. The core, representing an early time point, seems almost devoid of secondary structural elements such as protein beta-sheets and alpha-helices and RNA basepair helices. Ribosomal protein components near the PTC are indicative of peptide ancestors of ribosomal proteins. Length restrictions did not allow them protein secondary structure, which is almost absent from the PTC-origin. However, such structures steadily increase over time. The evolution of the early PTC also made use of Mg^2+^ ions (but see below) for coordination of partially single-stranded RNA species, which are replaced by proteins in periphery. The fact that we do not find “paleontological traces” of amino acids or tiny peptides in the center does not mean they were not involved in protection against breakdown (see previous paragraphs) or assisting folding of the “precursor” RNA. However, it does strongly support the notion that catalysis of peptide formation is achieved by RNA *only*. The role of amino acids and di- or tripeptides would thus have been restricted to protection of this primordial RNA against breakdown. This is still essential, however, as the presence of Mg^2+^ ions is a mixed blessing. These ions cannot only coordinate RNA, but can also catalyze hydrolysis [66], unless chelated by citrate [67] or short acidic peptides [10,67]. This hydrolysis is even more efficient in the case of single stranded RNA as observed to be present in the ancient core. Before the primordial channel could interact with “extremely short RNA chains carrying amino acids, and possibly di- or tripeptides” [62] these “activated” forms would have to appear. This, in turn, would imply that the tiny RNA center would be present in a milieu of abundant precursors of such molecules, the chemically versatile amino acids themselves. All these considerations make the early coevolution of the “PTC-tunnel core” with amino acids likely. This could even imply that peptides made by this protoribosomal core may have become able to replicate RNA (see the next section) before possible RNA-based RNA replicases ever evolved. This brings us to an ancient highly conserved protein RNA replicase, characteristics of which make this scenario not unlikely: RNA polymerase.

## 13. Evolutionary Important Characteristics of RNA Polymerase

We have arrived at a juncture where (partially) coded limited peptide synthesis seems up and running. If these small peptides could boost RNA synthesis, the precursors for present-day mRNA molecules could be generated. The last of the RNA trinity (tRNA, mRNA, and rRNA) could thus come about. The ancient, crucial, tripeptide AspGlyAsp most likely chelates Mg^2+^ to protect the RNAs from breakdown. Sequence comparison shows it to be the sequence, which evolved (upon extension in the “PTC-tunnel core”) to replicate RNA (probably in cooperation with RNA and Mg^2+^). AspGlyAsp evolves into AspAlaAspValAspGlyAsp, (DADVDGD), being the obvious ancestor sequence of the universal RNA polymerase active site sequence NADFDGD. Of this sequence, the three aspartic acids interspaced by a bulky residue and a glycine seem to be a universal RNA polymerase signature, e.g., of both the enzyme that produces mRNA with DNA as the template as well as of the enzyme that makes microRNAs with RNA as template. The pentapeptide DVDGD or its heptapeptide extension on their own are without known catalytic polymerase activity [35]. In crystal structures the universal motif DbDGD (b represents a bulky residue), binds to RNA with the aid of a coordinated divalent cation, such as Mg^2+^. It is highly likely that this sequence formed a crucial part of the oldest RNA replicases, whether they were more based on RNA or on protein for their remainder. Such interactions, where “acid amino acid rich” peptides or even simpler di- and tri-carboxylic acids would coordinate Mg^2+^ to protect RNA, minimize wobble mismatch formation, and in general to enhance chances of some kind of RNA replication to evolve, have most likely been crucial in early evolution [10].

## 14. A Proposal for the First Stages of Genetic Code Development

We are now in a position to describe a possible scenario for the first stages in genetic code development. We start out with stem-loop tRNA predecessors that can be charged by whatever amino acid is present, most likely mainly Glycine, Alanine, Aspartic acid and Valine. In this situation, tRNA-tRNA interactions can produce dipeptides, among which GlyGly would predominate. The presence of “Shimizu-like” C4N structures would allow even further increases in the amount of GlyGly dipeptides. Their presence would increase the chances of the development of small ribozymes such as the precursor of the large rRNA. Variations in C4N structures would give the possibility to expand the number of specifically loaded tRNAs to three next: Alanine and Valine would join Glycine. The fourth variant tRNA precursor is still incorporating activated amino acids non-selectively. Among these, Aspartic acid will be of major importance. During this development small RNA stem-loop molecules interact with each other to produce cloverleaf tRNA precursors (according to Di Giulio’s major insight). This produces an anticodon at the “right side” (Figure 1), and explains links between anticodon sequences and RNA binding preferences for certain amino acids. The protective, stabilizing aspect of Aspartic acid on RNA makes it possible to produce Aspartic acid containing peptides in a semi-coded way (via a positive feedback loop on protected RNA molecules that would enhance Aspartic acid loading). The precursor of large rRNA will interact with the loaded tRNA precursors in its evolving cavity and allows peptide synthesis to produce longer oligopeptides. Longer acidic peptides in turn would protect RNA against Mg^2+^. The ribozyme world evolves in *this* context: Reigning peptides are made up of Glycine, Alanine, Aspartic acid and Valine. Despite being very small, they (e.g., ValAsp, AspGlyAsp and GlyGly) already do have rudimentary enzymatic functions [4,10,18,36]. In this situation an RNA replicase/polymerase evolves. Tiny RNA fragments thus are synthesized more effectively and form the “mRNA” precursors. In this context also the precursor of the small rRNA appears. It possibly was selected for protection of the “mRNA” precursors first. The appearance of the two rRNA precursors and “mRNA” molecules both speeds up the process of peptide synthesis and enhances more specific termination by hydrolysis. Almost as a side-effect coded synthesis appears: Now RNA’s that encode small peptides that strengthen the complex and some of its emerging functions will be selected. Probably a 100-nucleotide ribozyme polymerase never existed.

## 15. Further Developments in Termination

At the stage just described, water, the availability of loaded tRNA precursors and “rRNA” were the only factors involved in termination, giving very limited options for improvements in both speed and specificity of the process. At a later stage *release factors* emerged. Originally the tRNA (unloaded, in what was to become the A-site) could catalyze “termination” itself (*i.e.*, it was the original release factor). Such termination of the peptidyl-tRNA by hydrolysis of the ester bond would occur in the precursor of the P-site. This mechanism has been mentioned by Noller [12] citing [68,69,70,71,72]. Our suggestion is that first, *every unloaded* tRNA (in the phase characterized by peptides containing Glycine, Alanine, Aspartic acid and Valine) had the ability to terminate further peptide bond formation. In this way, peptide synthesis was aborted in case of amino acid shortage. Later a specialized tRNA would be able to catalyze termination, the one with anticodon CUA being the obvious candidate. This, however, is in a rather advanced stage, when more amino acids are in the repertoire than just Glycine, Alanine, Aspartic acid and Valine. We find it quite amazing that the possible RNA activity in termination is not getting more attention in the research community, as illustrated by the paucity of citations of articles reporting termination activity of A-site unloaded tRNAs.

## 16. Conclusions and Discussion

We have suggested an intricate relation between the world of small RNAs and tiny “preproteins” (peptides containing Glycine, Alanine, Aspartic acid and Valine), the emerging ribosome, and the earliest synthetases that replaced ValAsp and “ribozyme synthetases” in the emerging translational system. For Glycine, Alanine, Aspartic acid and Valine, ribozymatic mechanisms existed to load the tRNAs. However, one has to think in terms of “Shimizu interactions” as described above (and of which the model studies should be urgently revisited and extended), and feedback mechanisms, such as “soak-it in Aspartic acid” strategies. Long ribozymes doing complex things were not a part of the early living world. Among the first functions possibly taken up by preproteins were peptide production (GlyGly), aminoacylation (ValAsp), RNA replication (AspGlyAsp), ribosome formation (r-preproteins), and more specific aminoacylation (Class 1 and Class 2 synthetase couples). During the era of coupled synthetases, three of such couples emerged: The couple distinguishing aliphatic, hydrophobic (Valine, Isoleucine, Leucine) from small (Serine, Proline, Threonine); the couple doing the same for Glutamic acid and its counterpart Aspartic acid; and the couple handling Tyrosine and Phenylalanine. These couples of course coincide with the synthetase subclasses. The last couple, however, emerges after the “Glycine, Alanine, Aspartic acid and Valine world” has been replaced by a more diverse protein world. These later stages of the history of life do not fall within the scope of this article. The original coupled function of synthetases has left its mark on the structure of the genetic code (among many other factors which have left their imprints behind in this structure; see above). One of our conclusions regarding the early RNA world must be: Long complicated ribozymes performing highly complex tasks were probably not part of the early living world (see also [10]).

In our article we did not discuss some very important other aspects of early evolution. In support of our considerations we find that of all the RNA species found in living organisms today, only the ones directly involved in translation (the process intimately linking amino acids and RNA from early on in evolution) are consistently found highly conserved in all three domains of life: tRNA, rRNA, and mRNA. Practically all other RNA molecules, e.g., involved in posttranscriptional processing, regulation and DNA replication are not found in all domains [73,74]. There are a few interesting exceptions: RnaseP (which cleaves off precursor sequences of tRNA molecules) *is* found in all domains of life. It is indirectly involved in translation. tmRNA, which rescues stalled ribosomes and thus is directly involved in translation, is only found in eubacteria and some mitochondria [75,76]. SRP RNA, however, forms the most interesting exception. It is crucial for the recognition and co-translational secretion of specific proteins (those having leader sequences) over cellular membranes. Thus, it is also “indirectly” involved in translation. This highly conserved RNA *is* found in all three domains. Its universality in living forms reminds us of the fact that translation probably began to evolve in the presence of membranes. Lipid (like) membranes already segregated the early biochemistry of life. Membranes were instrumental in maintaining and protecting high concentrations of precursors and products. Again, this function could be a mixed blessing, as dead end products or interfering molecules could also be trapped. As such, membranes remind us of the microenvironments at the interphase of different earth systems discussed above, which have to concentrate and combine while retaining flexibility to allow further processes. The “flexibility” of membranes in this era had to come from non-protein channels, possibly consisting of polyhydroxybutyrates and/or inorganic polyphosphates [77]. Here we might see another illustration of our overarching theme: Phosphates, important in activating precursor tRNAs could also have been important in primordial membrane channels; citrate, central in metabolism can also protect RNA; and, last but not least: RNA and amino acids protect each other and allow one another to more efficiently form polymers. Life is an ongoing exercise in systems biology.
